# Acute and Chronic Pancreatic Inflammation

**DOI:** 10.1155/2012/481658

**Published:** 2013-01-03

**Authors:** Derek A. O'Reilly, Zoltan Rakonczay, Marja-Leena Kylänpää

**Affiliations:** ^1^Department of Surgery, North Manchester General Hospital, Delaunays Road, Manchester M8 5RB, UK; ^2^Honorary Senior Lecturer, University of Manchester, Manchester, UK; ^3^First Department of Medicine, University of Szeged, P.O. Box 427, 6701 Szeged, Hungary; ^4^Second Department of Surgery, Helsinki University Central Hospital, Haartmaninkatu 4, 00290 Helsinki, Finland

Acute and chronic pancreatitis result in considerable morbidity, are increasing in incidence, and have a high mortality rate. Due to the inaccessibility of the pancreas to study, our understanding of the pathophysiology of pancreatitis remains limited. Our current knowledge of the evolution of pancreatitis can be described as a progression from an initial injury to both the acinar and ductal components of the exocrine pancreas to local and systemic inflammatory responses [1]. If resolution fails to occur, infection or chronicity may supervene. In this special issue we present a series of review and original papers that shed light both on our evolving understanding of the basic mechanisms causing pancreatitis as well as current treatment controversies.

**Lessons about Basic Mechanisms**

Toll-like receptors (TLR) belong to the superfamily of interleukin-1 receptors and enable the innate immune system to recognize different groups of pathogens, while initiating appropriate and distinct immunological responses. TLR4 proteins, expressed on the cell surface, are receptors for the Gram-negative bacteria cell membrane component, lipopolysaccharide (LPS, endotoxin). The heat shock protein HSP60 may play an important role in the protection of the pancreas against damage and against early zymogen activation in acute pancreatitis. J. Bonior et al. report a series of experiments investigating the effects of endotoxemia induced in newborn rats on TLR4, HSP60 and proapoptotic Bax, caspase-9 and -3, or antiapoptotic Bcl-2 protein expression in the pancreatic acinar cells of adult animals. Their results indicate that exposure of the infant rats to LPS promote the induction of HSP60 via TLR4 in their adult life and, in turn, activated Bax/Bcl-2 and caspase-9 and -3. It is likely that this process could play a part in the LPS-induced protection of the pancreatic tissue against acute damage in this experimental model.

Melatonin, a product of the pineal gland, is released from the gut mucosa in response to food ingestion and specific receptors for melatonin have been detected in many gastrointestinal tissues including the pancreas. Melatonin has been shown to attenuate the severity of acute pancreatitis. J. Jaworek et al. review the protective effects of melatonin on acute pancreatitis, reported in many experimental studies and supported by clinical observations. They conclude that the beneficial effects of melatonin are sufficient to warrant clinical evaluation as a supportive therapy in acute pancreatitis.

The causal relationship between tissue injury and pancreatitis and the mechanisms whereby tissue injury induces pancreatic inflammation is examined in a series of experiments reported by Nakumura et al. Although DNA has historically been believed to be immunologically inert, it is now appreciated that DNA can be released into the systemic circulation when cells undergo necrosis/apoptosis and recognized by the immune system. These authors hypothesised that cytosolic double-stranded DNA released by injured host cells may act as a “danger signal,” which affects pancreatic stellate cells by increasing the expression of several inflammatory genes in the rat.

Research into the pathogenesis of pancreatitis is hampered by the inaccessibility and hazards of pancreatic tissue sampling during the course of the disease as well as by the ethical constraint that pancreatic tissue sampling is not a routine part of disease management. Infected pancreatic necrosis (and some cases of sterile necrosis) is an indication for intervention and I. Kovalska et al. have put together a remarkable series of 224 operative and postmortem specimens subjected to histological analysis. This study underlines the importance of early pancreatic microcirculation and local coagulation disorders in the pathogenesis of acute necrotising pancreatitis and that, despite aggressive surrounding necrosis, the islets of Langerhans are preserved in 74.1% of cases, most probably due to fibrin capsule formation. This emphasises the importance of the movement in modern surgery to undertake removal of necrotic pancreas using minimally invasive and organ-preserving approaches [2].

Bridging the gap from bench to bedside, the editors of this special issue review the pro- and anti-inflammatory mediators that drive acute pancreatitis. This review examines the prospects for inflammatory mediators being identified as successful therapeutic targets. We explore the role of immunomodulation, monitoring the state of immune dysfunction by monocyte HLA-DR, signalling pathways of circulating leukocytes, and the narrow therapeutic window for intervention in acute pancreatitis.

**New Insights into Current Treatment**

The search for a specific therapy to treat pancreatitis remains the paramount goal of research in this field. Despite extensive efforts so far, no agent has proved successful. Further efforts to mount a therapeutic damage control strategy to contain an inappropriate inflammatory response are justified and remain ongoing. The APCAP (activated protein C in acute pancreatitis) trial randomised 32 patients with severe acute pancreatitis to receive either recombinant activated protein C (drotrecogin alfa activated) or placebo for 96 hours [3]. The results of the intervention and placebo groups were comparable. However, trials such as this also provide the opportunity to examine the systemic inflammatory response in great detail as it modulates with time and according to treatment. In this issue, L. Kyhälä et al. present the time course of the patients' plasma or serum levels of soluble markers (IL-8, IL-6, IL-10, IL-1ra, sE-selectin, PCT) and monocyte and neutrophil cell surface (CD11b, CD14, CD62L, HLA-DR) markers of systemic inflammatory response during the first 14 days after the randomization within APCAP.

With an increasing elderly population in many parts of the world, the effect of ageing on systemic inflammation, focusing on that induced by acute pancreatitis, has become more important than ever. Several reports have related an increased susceptibility to a proinflammatory status called inflammaging, which decreases the capacity of the immunological system to respond to antigens. M.C.C. Machado et al. review the effects of aging on the systemic inflammation in pancreatitis. They discuss the effects of cellular senescence and how this probably contributes to the chronic inflammatory state related to ageing.

State-of-the-art imaging remains a cornerstone of current management in acute pancreatitis. Early diagnosis and staging of complications are important clinical objectives. In this special issue, the method by which perfusion to an organ is measured by CT, and its clinical utility is reviewed by Y. Tsuji et al. They make the case that perfusion CT is a promising technique for diagnosis of local and systemic complications of severe acute pancreatitis at an early stage. Discriminating between focal chronic pancreatitis and pancreatic cancer remains challenging. Contrast-enhanced endoscopic ultrasound using Doppler techniques can reveal different vascularisation patterns in pancreatic tissue altered by chronic inflammatory processes and pancreatic cancer. M. Hocke et al. review the basics of contrast-enhanced high and low mechanical index endoscopic ultrasound and explain the pathophysiological differences behind the vascularisation of chronic pancreatitis and pancreatic carcinoma and how to use these techniques in daily clinical practice.

Pancreatic pseudocyst is a complication that develops in both acute and chronic pancreatitis. It may remain asymptomatic or develop life-threatening complications. A. K. Khanna et al. address the therapeutic dilemma of whether or not to treat a patient with a pancreatic pseudocyst, as well as when and with which technique.

**Conclusion: From Early Accounts to Future Hopes**

An early account of acute pancreatitis may have been furnished by the death of Alexander the Great** (**Alexander III of Macedon (20/21 July 356–10/11 June 323 BCE)). Alexander ascended to the throne and was to become the most successful of Greek generals, extending Hellenic influence throughout the known world. However, a case for acute pancreatitis being retrospectively applied to Alexander's death certificate can be made: a rich meal and heavy alcohol consumption preceded the onset of the disease and were probably the precipitating factors; the course of the disease is typical of acute pancreatitis in its onset, severity and irreversibility; fever and the subsequent systemic effects point towards acute necrotising pancreatitis with sepsis and multiple-organ failure [4]. If the cause of death was indeed acute pancreatitis, then perhaps the chief lesson to be learnt from this account is to reinforce the fact of our lack of progress in our understanding and particularly, in treating this condition. It should be remembered that (despite recent advances in critical care medicine) no specific treatment for acute pancreatitis has, as yet, proved superior to that employed by Alexander's generals; a forlorn plea for divine intervention, to the gods of the ancient Greeks.

We hope, however, that ongoing research, such as that contained within this special issue of the International Journal of Inflammation, provides the insight and inspiration necessary to make progress in our endeavours to obtain sufficient understanding to discover an effective treatment for this disease.
